# Dyspepsia and Drunkenness

**Published:** 1859-10

**Authors:** 


### DYSPEPSIA AND DRUNKENNESS.

A DRUNKARD is never so great a fool as to kill himself; the dyspeptic is.

More persons are destroyed by eating too much, than by drinking too much. Gluttony kills more than drunkenness in civilized society.

The dyspeptic kills himself; the drunkard kills others.

The dyspeptic takes his own life under the influence of mental depression; the drunkard kills others under the influence of mental excitement. But, although both are alike unconscious at the time of what they are doing—one slaying himself, the other slaying his fellow-man—the suicide has the sympathies of society, and finds among it many apologists; while towards the drunken murderer of another, the feeling is one of vindictive impatience for the gallows to do its duty.

Both the drunkard and the dyspeptic are unconscious of crime at the instant of its perpetration. Both states are brought on by over indulgence of the appetite; the one for food, the other for drink; and both end in shedding blood.

The dyspeptic lays his plans for self-murder with deliberation; the drunkard murders another in the surprise of ungovernable passion; and, if deliberation darkens the deed, then is the drunkard the less criminal of the two.

If the drunkard is murderously inclined, it is only for a brief hour, while the fit is upon him, and he need be watched only for that time. But the dyspeptic, who is set on his own heart's blood, must be watched sedulously for days and months, or, the first moment that the eye is off his movements, he improves to his ruin.

Few palliate the drunkard's deed, while the dyspeptic meets with universal sympathy. Should this be so? What is the ground for this partiality? Surely all are called upon to mature this subject and to inquire with a feeling of considerable personal responsibility, if, in the matter of eating, there is a daily watch against excesses, which so often end in that worst of all crimes, (because done with deliberation, and is not repented of) self-murder!

